# Quantitative Understanding of Ionic Channel Network Variation in Nafion with Hydration Using Current Sensing Atomic Force Microscopy

**DOI:** 10.3390/polym16050604

**Published:** 2024-02-22

**Authors:** Osung Kwon, Jihoon Lee, Hyungju Son, Jaehyoung Park

**Affiliations:** 1 Faculty of Science, Tabula Rasa College, Keimyung University in Seongseo, Daegu 42601, Republic of Korea; 11502@gw.kmu.ac.kr; 2 AET Co., Ltd., Daegu 41967, Republic of Korea; ricky.lee@aetechnology.co.kr (J.L.); edward.son@aetechnology.co.kr (H.S.)

**Keywords:** ionic channel network, proton conductivity, proton exchange membrane, proton exchange membrane fuel cell, current sensing atomic force microscopy, atomic force microscopy

## Abstract

Proton exchange membranes are an essential component of proton-exchange membrane fuel cells (PEMFC). Their performance is directly related to the development of ionic channel networks through hydration. Current sensing atomic force microscopy (CSAFM) can map the local conductance and morphology of a sample surface with sub-nano resolution simultaneously by applying a bias voltage between the conducting tip and sample holder. In this study, the ionic channel network variation of Nafion by hydration has been quantitatively characterized based on the basic principles of electrodynamics and CSAFM. A nano-sized PEMFC has been created using a Pt-coated tip of CSAFM and one side Pt-coated Nafion, and studied under different relative humidity (RH) conditions. The results have been systematically analyzed. First, the morphology of PEMFC under each RH has been studied using line profile and surface roughness. Second, the CSAFM image has been analyzed statistically through the peak value and full-width half-maximum of the histograms. Third, the number of protons moving through the ionic channel network (NPMI) has been derived and used to understand ionic channel network variation by hydration. This study develops a quantitative method to comprehend variations in the ionic channel network by calculating the movement of protons into the ionic channel network based on CSAFM images. To verify the method, a comparison is made between the NPMI and the changes in proton conductivity under different RH conditions and it reveals a good agreement. This developed method can offer a quantitative approach for characterizing the morphological structure of PEM. Also, it can provide a quantitative tool for interpretating CSAFM images.

## 1. Introduction

Among various green energy devices, the proton-exchange membrane fuel cell (PEMFC) is a promising energy conversion device for reducing carbon dioxide in the air. PEMFC has a high energy density and energy conversion rate [1]. Also, it can generate a few watts to a few hundred kilowatts of electrical power. Thus, PEMFC can be utilized in a wide range of applications, from mobile devices to microgrids [2,3,4,5]. The proton-exchange membrane (PEM) is a crucial component of PEMFC. PEMs work as gas separators, insulators between the anode and cathode, and proton conductors. 

Nafion is one of the successfully commercialized PEMs due to its good chemical and mechanical properties. Also, it has good proton conductivity. Such extraordinary characteristics of Nafion are attributed to its unique morphological structure. Nafion is synthesized by adding sulfonic acid side chains to a polytetrafluoroethylene (PTFE) backbone. Thus, the morphology of Nafion depicts two different phases corresponding to the hydrophobic PTFE backbone and hydrophilic SO_3_^−^ side chain. Since the sulfonated side chains are highly hydrophilic, one end of the molecules mixes with water. Water supply to the hydrophilic region near the sulfonic acid groups is generated by hydration on the membrane. In this region, easily removable protons are weakly attached to the sulfonic acid groups. This phase separation on Nafion is a source of proton conductivity. Thus, multiple studies for understanding the morphology of Nafion have been undertaken by many groups [6,7,8,9,10]. One of the widely accepted morphological models of Nafion is the cluster-network model. Gierke et al. studied the morphological structure of Nafion through small angle X-ray scattering and wide-angle X-ray scattering (WSRS) [6]. 

**Figure 1:**
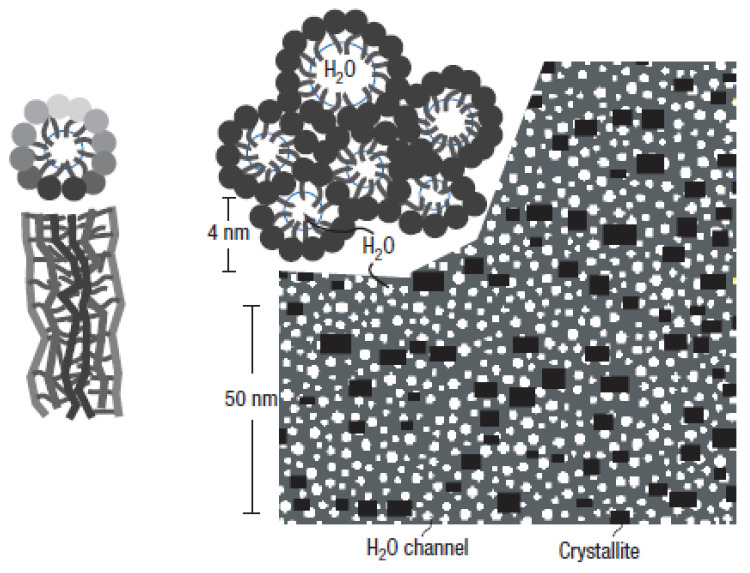
Schematic of the cylindrical water channel model [11].

In the cluster-network model, ionic clusters with an inverted micellar structure have a spherical shape with a diameter of 4 nm. Each ionic cluster is connected by 1 nm narrow water channels. Under hydration, the channel forms a more complicated network and protons move through the network. Thus, proton conductivity is directly related to the ionic channel network. Klaus and Chen proposed a cylindrical water channel model, which is based on simulations using previous scattering studies [11]. This model accounts for the good proton conductivity and mechanical properties of Nafion well. Figure 1 illustrates a schematic of the cylindrical water channel model. Nafion contains crystallites and cylindrical water channels of radius 2–3 nm. The cylindrical water channel is a source of proton conductivity and its volume increases with hydration. Crystallites are exposed in the membrane and they provide mechanical strength to Nafion. 

Understanding the ionic channel network is not easy, even though remarkable morphological structures of Nafion, such as the cluster-network and water channel models, have been proposed based on the scattering and simulation studies because the ionic channel network varies under hydration. Thus, the in situ measurements of Nafion structure are among the best ways to understand the ionic channel network. Atomic force microcopy (AFM) possesses great potential to study the ionic channel network on Nafion because it can measure thermal, electrical, and magnetic properties on the membrane [11,12,13]. Among various extended modes of AFM, electrostatic force microscopy (EFM) and current sensing atomic force microscopy (CSAFM) are widely used. 

EFM is typically used for the electrical characterization of a sample surface [14,15,16,17]. EFM, which uses a vibrating tip, can map local charge distributions on the surface in the form of a phase lag value distribution. For heterogeneous materials, which have separate conducting and non-conducting regions, EFM is an effective tool to study the surface morphology and electrical characteristics. Thus, EFM is considered to be a proper tool for the characterization of the ionic channel network of Nafion. Austin M. Barnes and Steven K. Buratto studied the imaging channel connectivity of Nafion [18]. The ionic channel connectivity in the proton exchange membrane is directly correlated to its performance. They characterized various ionic channel shapes based on the quantitative analysis of the EFM signal. Shizheng Yi et al. [19] proposed a novel PEM based on sulfonated poly(ether ether ketone) and imidazolium-type ionic liquids for high-temperature PEMFC [19]. By using AFM and EFM, the morphology of the ionic channel network was analyzed and the results showed a clear phase separation of hydrophilic and hydrophobic regions along with a stable performance of the PEM near 349 °C. Phosphonium-containing diblock copolymer anion-exchange membranes were studied by Austin M. Barnes et al. [20]. The connectivity of channels was characterized using AFM and EFM. For different ion exchange capacities, different channel alignments were observed, such as those parallel to the surface and perpendicularly aligned channels. With IEC = 0.44 mmol/g, cylindrical channels were aligned parallel to the surface and disconnected regions were observed. When IEC was 0.87 mmol/g, perpendicularly aligned channels were observed and the ionic phase was connected well throughout the membrane. PEM studies with EFM revealed meaningful results for the characterization of the ionic channel network; however, it is insufficient to understand the morphology of PEM because observation with operating PEM is required for detailed understanding. 

CSAFM can characterize local conducting properties by using current variation on the surface. For this, a sample is placed between the conducting tip and a metal plate, and an extremely weak current is sensed by applying DC voltage. This method has great potential to characterize the ionic channel network of PEM as it allows in situ measurements of the structure by creating nano-sized PEMFC, which consists of a Pt-coated tip, PEM, Pt catalyst, and gas diffusion layer. Thus, many studies for understanding the morphology of PEM have been performed by several groups using CSAFM [21,22,23]. Xiaojiang Wang et al. utilized CSAFM and electrochemical impedance spectroscopy for recording conducting data of the three-phase boundary [21]. The localized proton-transport resistance was measured under different relative humidity (RH) conditions. Also, the spatial diversity of proton-transport resistance was observed in the uncertain concentration of the hydrophilic region. Qinggang He and Xiaoming Ren studied the ion conductance variation of the anion-exchange membrane (AEM) surface using CSAFM [22]. They discovered a disparity between the proton-exchange property and ionic conductance. The proton-exchange property was found to be one order higher in magnitude than the ionic conductance of the membrane under 100% RH due to the rate-limiting properties of AEM. When RH was lower than 45%, the opposite result was observed. The surface conductance variation of Nafion due to aging was characterized using CSAFM by Zhu’s research group [23]. In this study, Nafion was annealed in order to accelerate aging and surface conduction distribution was characterized. The results show that a clear separation of conducting and non-conducting regions appeared due to aging, and also a conducting area rotation was observed by annealing. 

Several studies have been carried out to understand the morphology of PEM using conducting AFM techniques such as EFM and CSAFM [24,25,26]. Heisgen et al. [25] studied the correlation of conductivity with other mechanical properties of PEM. They simultaneously measured the surface conductance and mechanical properties of PEM using tapping mode AFM and CSAFM. From these measurements, they analyzed the water and current flows in PEM. Masanori Hara et al. [26] characterized the conductive area of a PEM using CSAFM and measured the conductive spots on the membrane. They demonstrated that the reversible and irreversible changes observed in the conductive area were more than necessary, and these changes were related to the rearrangement of the proton conducting path and performance enhancement, respectively. Therefore, these studies provided remarkable results for understanding the ionic structure on the PEM. However, most of these studies have focused on the qualitative understanding of the ionic domain or ionic channel network based on the morphological characteristics of the PEM. Furthermore, they have focused on characterizing changes in the ionic structure based on the conductance distribution on the PEM surface. Therefore, quantitative information is inadequate to understand the ionic structure variations on the PEM. Additionally, the quantitative studies of the ionic channel network variation based on CSAFM are insufficient to understand the morphology of PEM. 

Hence, this study aims to accurately and systematically investigate the ionic channel on the PEM based on the quantitative analysis using current distribution. To this end, a numerical approximation model is derived based on the basic principles of electrodynamics, CSAFM, and electrochemical activity in the PEM. Moreover, the ionic channel density variation is analyzed based on the numerical approximation model, while the result of the quantitative analysis indicates that the PEM underwent hydration. Herein, CSAFM is used to understand the ionic channel network based on the ionic activity on the Nafion surface due to hydration. The study is conducted accurately and systematically using multiple steps. First, topography and CSAFM images at elevated RH are interpreted using qualitative and statistical methods. Second, a simple numerical approximation model is derived from the basic principles of electrodynamics, CSAFM, and PEM. Finally, current values from CSAFM are analyzed to understand the ionic channel network variation due to hydration based on the simple model for driving proton movement into the membrane.

## 2. Experimental Setup

**Figure 2:**
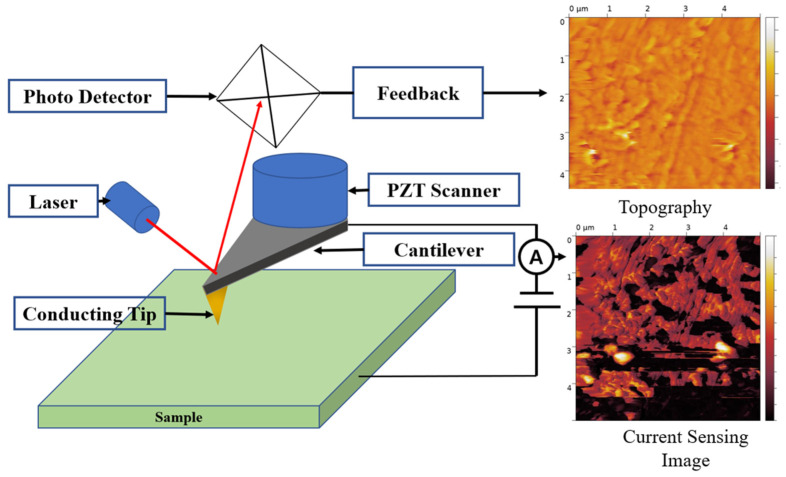
Schematic of CSAFM.

In this study, CSAFM has been used for measuring the surface current distribution of the membrane. CSAFM is an extended technique of AFM and can simultaneously map the topography and current distribution on the surface. Figure 2 shows the schematic of CSAFM. CSAFM can simultaneously detect multi-signals between a tip and the sample surface, such as atomic force and local current. When a conductive tip scans a surface by maintaining the applied bias voltage between a tip and the sample surface, the deflection of the cantilever is quantified by measuring a laser beam, which is reflected from the cantilever end to the position-sensitive photo detector, thereby generating the topography on the scanned surface. Meanwhile, a conductive tip senses the current flow between the tip contact and the plane electrode on the other side of the membrane, generating current-sensing images. The working of the CSAFM involves recording a sensitive electric current loop which connects a conducting probe tip and the sample. For sensing an extremely weak surface current, a conventional AFM tip uniformly coated with a metal layer is used as a sensor. When the conducting tip scans over the membrane surface in contact with the sample surface, it traces out the surface morphology and measures the current flow at the pointed position simultaneously. 

**Figure 3:**
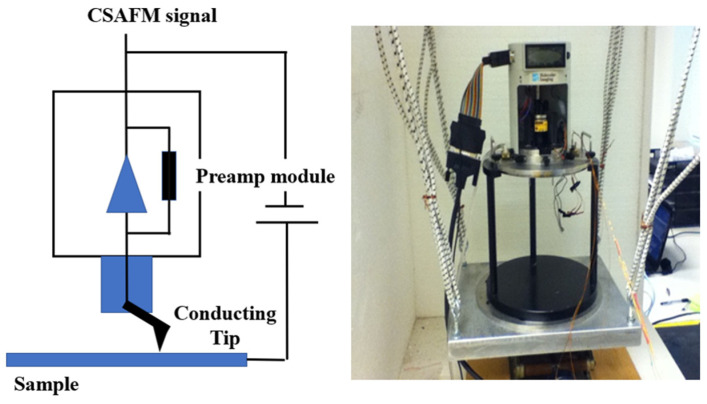
Schematic of the CSAFM-based measuring system used in this study.

Figure 3 depicts a simple schematic of the measuring system used in this study. The system consists of an environmental chamber and the measuring system. CSAFM is placed in the environmental chamber along with a heating and humidifying system that controls RH for hydration on the membrane. The heating system is used to evaporate water on the sample, so it is attached to the bottom of the conducting sample holder. The humidifying system supplies water vapor in the environmental chamber. The tip used in this study is uniformly coated with platinum. The sensitivity of CSAFM is from 10^−12^ A to 10^−8^ A.

**Figure 4:**
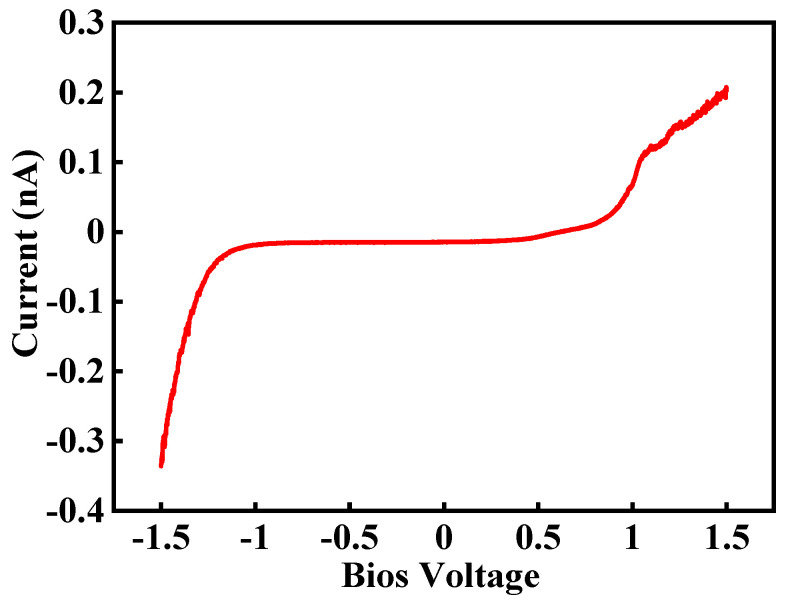
Current flow by swiping bias voltage from −1.5 V to +1.5 V [27].

Nafion 115 membranes purchased from Sigma-Aldrich (Seoul, South Korea) have been used in this study. The equivalent weight and thickness of the membrane are 1100 and 127 μm, respectively. For measuring proton movement into the membrane, half single cell has been created by using Nafion 115 and PtC coating on one side of the membrane. A Pt-coated tip is used for several purposes. First, it works as sensor for measuring weak current. Second, it is used for water electrolysis on the PEM. Third, it works as a catalysis layer for oxidizing hydrogen. Son et al. report current flow measurement by swiping the bias voltage from −1.5 V to 1.5 V, as shown in Figure 4 [27]. This result shows that the current increases considerably near to −1.5 V, and hydrolysis is increased and protons are generated when a Pt-coated tip is used.

This study involves several steps, such as sample preparation, measurements, and analysis. First, the membranes of size 1 cm × 1 cm are attached to the conducting sample holder and heated under 80 °C overnight in the environmental chamber to remove any moisture from the membrane. Next, the heater is turned off and Nafion 115 is cooled while maintaining the dry conditions for 2 h. 

**Figure 5:**
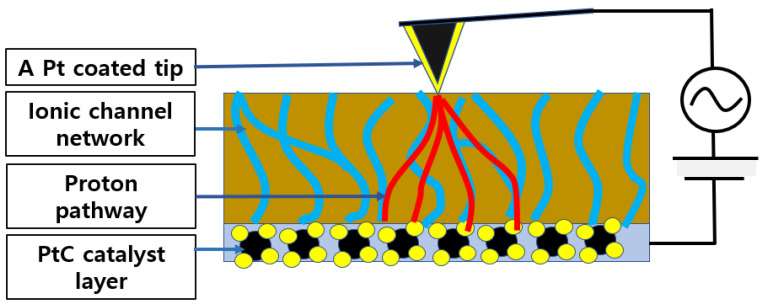
Nano-sized PEMFC based on CSAFM and Nafion 115 half single cell (Blue color line).

After preparation is finished, Nafion 115 is systematically and accurately measured using CSAFM. First, the Pt-coated tip approaches the half single cell made by Nafion 115, and the tip and cell create a nano-sized PEMFC, as shown in Figure 5. Second, the topography and CSAFM image is recorded by applying DC bias between the tip and the sample surface. Once the measurement is over, the tip is lifted up to detach it from the half single cell. The RH in the environmental chamber is raised by 10% and maintained for 2 h. The same measurement process is repeated again. This process is continued by increasing the RH in steps of 10% until an RH of 75% is reached. When the RH is higher than 75%, the sensitivity of CSAFM reaches its limit. 

The results have been interpreted using several methods. First, the topography at each RH has been analyzed for its line profile and root mean square (RMS) roughness. Second, CSAFM images at each RH have been characterized statistically using the peak value and full width half maximum (FWHM), which has been calculated from the histogram. Third, the images have been numerically studied by calculating the number of protons moving into the ionic channel network (NPMI) based on the basic principles of electrodynamics and CSAFM.

## 3. Experimental Results

**Figure 6:**
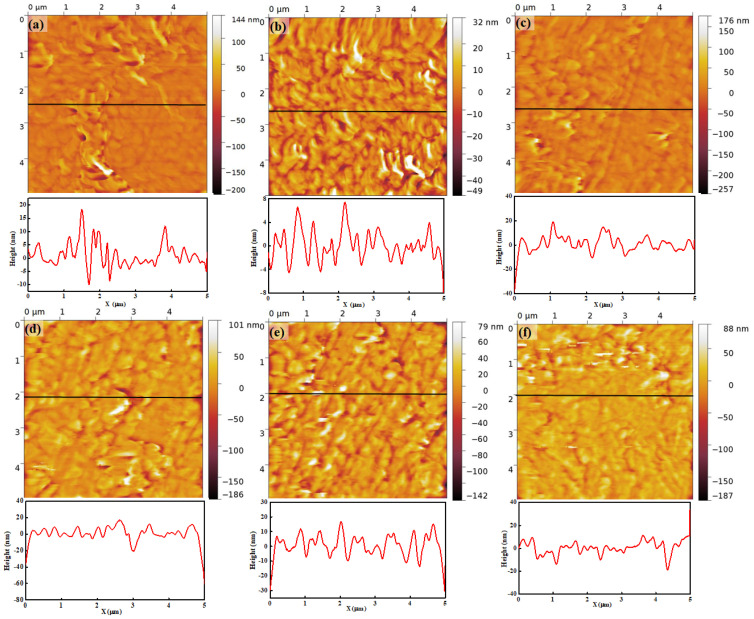
Topography and related line profiles of Nafion 115 under RH values of (**a**) 15%, (**b**) 25%, (**c**) 45%, (**d**) 55%, (**e**) 65%, and (**f**) 75%.

Figure 6 shows the topography of Nafion 115 under different RH levels. The scanned area of each image is 5 μm × 5 μm. The brightness of the color in each image indicates the height level on the surface, and the color bar placed on the left of the image shows the height level. Figure 6a–f show the morphology and relative line profile under 15%, 25%, 45%, 55%, 65%, and 75% RH, respectively. Each image shows random lumpy structures of sizes ranging from a few tens of nanometers to a few hundreds of nanometers on the surface. However, any remarkable morphological changes under increasing RH are not evident in the images. The line profile, which shows the height variation of a selected line, provides numerical information. At 15% RH, the height of the surface varies from −10 nm to 20 nm and it is relatively large compared with the height variation at a lower RH. This might be due to the protruded regions, which can be observed in the middle and top of the topography. The height variation is roughly less than 10 nm, excluding the protruded areas. At 25% RH, the height variation lies within 10 nm. At an RH higher than 45%, increases in the height variation can be observed in each case. There is no remarkable height variation difference from 45% and 75% RH and the magnitude of height variation is roughly from 40 nm to 60 nm. The height variation difference between low RH (15% and 25%) and high RH (45%, 55%, 65%, and 75%) is influenced by the water content in the membrane. The similar value of height variation under high RH might be due to insufficient hydration time. 

The information about surface morphology derived from the line profile does not provide detailed and accurate information. Alternatively, the surface roughness value is the widely accepted method to analyze surface morphology. The RMS roughness, which is the mean value of standard deviation from the average height of the topography, provides quantitative information about morphology. The RMS roughness is determined by Equation (1).

(1) $$R_{q}=\sqrt{\frac{\sum {\left(x_{i}-\bar{x}\right)}^{2}}{N}}$$

where *R_q_*, *x_i_*, $\bar{x}$, and *N* are RMS roughness, the height value of each pixel, the mean value of pixel height, and the number of pixels in the topography, respectively. Table 1 shows the RMS roughness for each RH value. At 17% RH, the RMS roughness is 18.3 nm, as calculated from the entire topography. The number within the bracket (7.0 nm) refers to the RMS roughness of the topography excluding the extruded region. At 27% RH, RMS roughness is 8.82 nm and it is similar to the RMS roughness at 17% RH. RMS roughness values at 45%, 55%, 65%, and 75% RH are 19.5 nm, 21.9 nm, 20.1 nm, and 21.4 nm, respectively. When RH is higher than 45%, RMS roughness is about three times larger than that at 17% and 27% RH. Under higher RH, RMS roughness values are similar at each RH, with the mean value being 20.7 nm. In this study, contact mode AFM has been used for scanning. A constant force is applied to the membrane surface during scanning, due to which the membrane surface becomes deformed and the RMS roughness shows similar values even if the membrane is swollen. This RMS roughness variation under different RH is well correlated with the line profile variation. 

**Figure 7:**
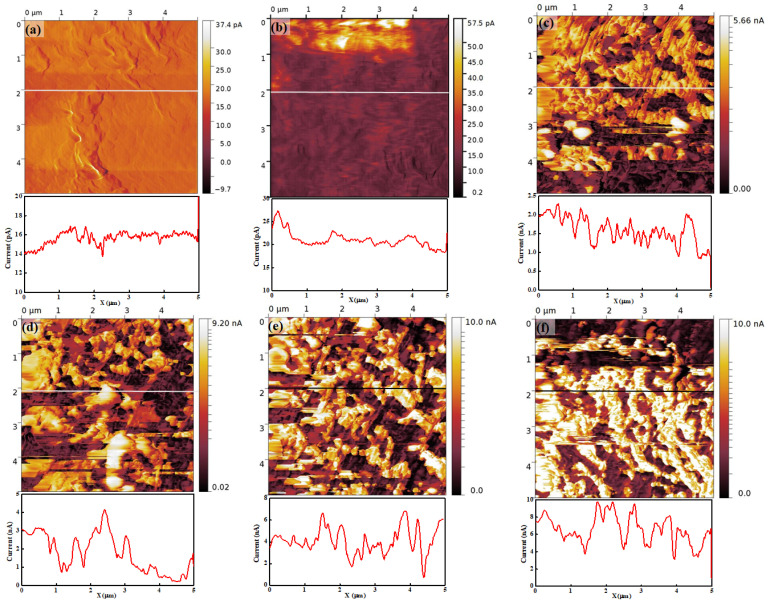
Current map and related line profile of Nafion 115 under RH values of (**a**) 15%, (**b**) 25%, (**c**) 45%, (**d**) 55%, (**e**) 65%, and (**f**) 75%.

Figure 7 is the current map of Nafion 115 under different RH conditions. The brightness of the current map represents magnitude and the color bar next to the current map shows the numerical value of current for each brightness. Figure 7a–f are the current maps and related line profiles of the membrane at 15%, 25%, 45%, 55%, 65%, and 75% RH, respectively. At 15% RH, the current map shows an extremely low current value distribution on the membrane surface. The current can be observed to vary within a few tens of picoamperes. Considering the presence of instrumental noise, which is typically a few tens of picoamperes, the measured current might be close to zero. Also, the current map has a structure similar to the topography in terms of protruded regions. This result implies that the current flow through the tip is extremely small and only the morphological structure on the surface is affected by it. At 25% RH, the current is slightly higher than the current at 17% RH, as shown in Figure 7b, and the current is about 20 pA, as derived from the related line profile. The top of the image, which appears as a brightly colored region, shows a relatively higher current. The current image also depicts the morphological structure such as the current image at 15% RH. It also implies that the current flow to the tip is extremely small at 25% RH. 

When the RH is higher than 45%, an entirely different current map can be observed compared with 15% and 25% RH. At 45% RH, the current increases by a hundred times compared with that at 25% RH. The current distribution on the membrane surface shows a clear separation of low and high current regions in the form of dark (low current) and bright (high current) regions. The high-current region corresponds to approximately 1.5 nA to 2.5 nA, and the low-current region corresponds to approximately 1 nA, as shown in the line profile in Figure 7c. Also, some completely black areas can be observed, where the current is smaller than the dark regions. The contrasting bright and dark regions create a crumbled shape. At other high RH conditions, the current distribution on the membrane surface shows a similar trend to that observed at less than 45% RH; for instance, a clear separation of low- and high-current regions. The current value in each region increases with increasing RH. The values in low current regions are approximately 1 nA to 2 nA, 2 nA to 4 nA, and 4 nA to 6 nA at 55%, 65%, and 75% RH, respectively. In high current region, the values are roughly 3 nA to 4 nA, 4 nA to 6 nA, and 8 nA to 9 nA at 55%, 65%, and 75% RH, respectively. The current on the membrane increases with increasing RH, and the distinct low and high current distributions can be observed from all current maps when RH is higher than 45%. The connectivity of the ionic channel, which is called the ionic channel network, increases and may lead to a diversification of the proton path through the hydration of the membrane. The current on the membrane increases because the hydration might progress by increasing the RH. 

**Figure 8:**
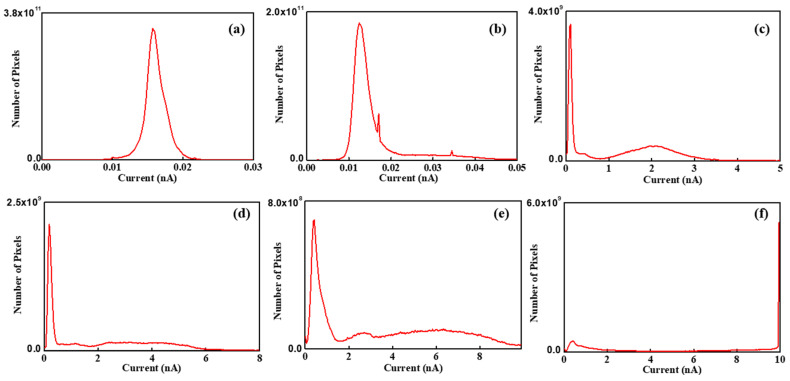
Histogram of current maps of Nafion 115 under RH values of (**a**) 15%, (**b**) 25%, (**c**) 45%, (**d**) 55%, (**e**) 65%, and (**f**) 75%.

For a more detailed understanding, a histogram of each image has been determined. The histogram can count the number of pixels, which denotes the total current at the area pointed by the tip, at each RH value. Thus, the peak value denotes the dominant current value on the membrane. Figure 8a is the histogram of the current map at 15% RH and shows a single peak near 15 pA. At 25% RH, also, the histogram shows a single peak near 15 nA. However, unlike at 15% RH, the pixels also exist from 20 pA to 50 pA and this indicates that a weak current flow exists between the tip and the membrane surface. From 45% to 75% RH, all histograms clearly show two distinct peaks. One peak appears near the low current, while another peak appears near the high current. On increasing RH, the height of the peak near the low current reduces continuously and this might be due to the pixels moving from the low-current to high-current region through hydration. The number of pixels of the second peak also increases with increasing RH. Second peaks of the histograms at 45%, 55%, 65%, and 75% RH correspond to approximately 2 nA, 4 nA, 6 nA, and 10 nA, respectively. This result is evidence of the increasing current flow between the tip and membrane surface, since the peak value represents the current between them. From 45% to 65% RH, the width of the second peak becomes broader continuously, as shown in Figure 8c–e. At 75% RH, the histogram shows similar features compared with other histograms, such as low and high current peaks; however, it shows a different detailed feature. At a low current peak, an extremely small number of pixels is placed, and at a high current peak, only one straight line can be observed, with the peak value of 10 nA. 

Table 2 lists the peak value and FWHM of each histogram. The first and second peak values are calculated near the low- and high-current region, respectively. Current flow is the movement of electrons corresponding to the hydrogen atoms. When electrons move towards the tip, the protons move into the ionic channel network. The peak value is proportional to the current flow between the tip and the membrane surface. It might be related to the mean ionic channel network density of each region. FWHM represents the ionic channel network distribution on the membrane. In low RH conditions, the peak value at 15% RH is 25% higher compared with that at 25% RH. Unlike peak value, the FWHM at 25% RH is two times greater than that at 15% RH. The relatively low peak value at 25% RH is due to the shape of histogram. The shape of the histogram at 15% RH shows normal distribution, but that at 25% RH is shifted towards a high current direction due to increased current distribution. Considering average current, the value at 25% RH is 50% higher than at 15% RH. 

**Figure 9:**
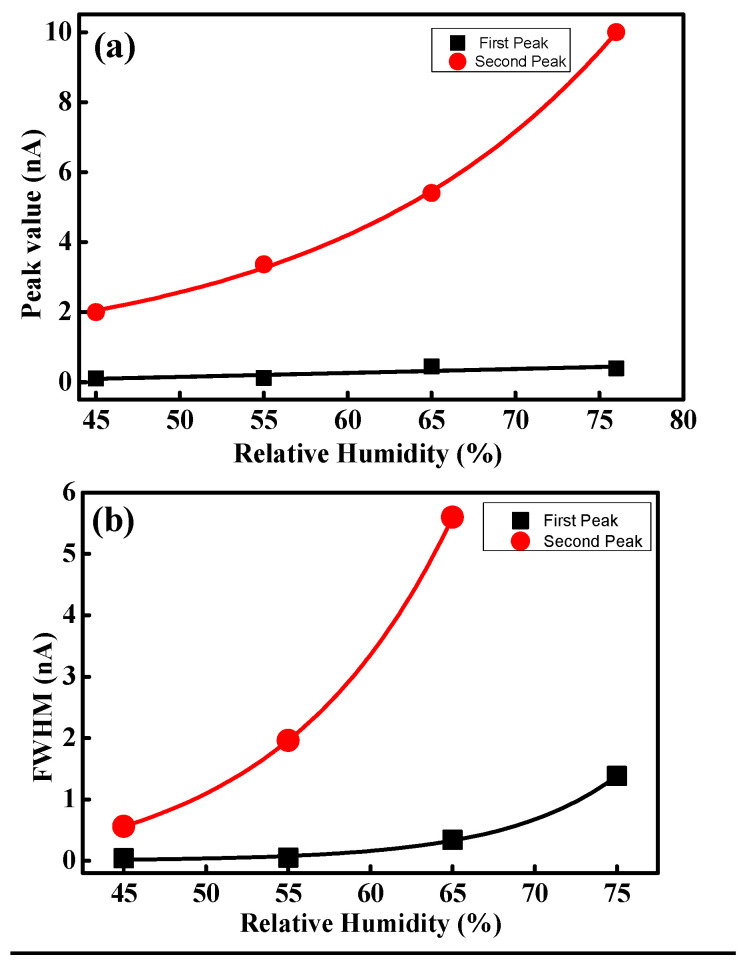
(**a**) Peak value and (**b**) FWHM variation under hydration.

At high RH condition, two peaks are evident in the histogram. Peak values and FWHM are plotted as shown in Figure 9 and fitted as an exponential function. The slope is linearized as log⁡y=log⁡A+x. The first peak value varies from 0.1 to 0.39 with increasing RH and the linearized slope is roughly 9 × 10^−4^ A^−1^. FWHM shows a drastic change, and the value at 75% RH is 35 times larger than at 15% RH. FWHMs vary from 0.04 nm to 1.38 nm and the linearized slope is about 0.09 A^−1^. The second peak values are 2.0 nm, 3.4 nm, 5.4 nm, and 10 nm at each high RH value from 45% to 75%, respectively. The corresponding linearized slope of the peak value is 0.06 A^−1^. The FWHMs of the second peak are 0.56 nm, 1.96 nm, and 5.6 nm at 45%, 55%, and 65% RH, respectively. FWHM at 75% RH cannot be calculated because all pixels which have high current are counted as 10 nA. This is due to the resolution limit of CSAFM, i.e., the maximum measurement current is 10 nA. The linearized slope of FWHM at the second peak is 0.14 nA. Comparing both peaks, it can be concluded that the linearized slope of the second peak value and corresponding FWHM is 66 and 1.5 times higher than that of first peak value, respectively.

## 4. Analysis

The peak value corresponding to the high-current region is directly connected to the ionic channel network. It is the mean value of the current flow between the tip and the membrane surface at each region. The current flow is proportional to the number of protons moving into the ionic channel network, which results from the oxidation of hydrogen. The magnitude of the current flow is related to the ionic channel network density. Thus, the peak value represents ionic channel network density in the membrane. Two peaks appeared in the histogram of the ionic channel network, as shown in Figure 8. The first peak values showed small variation with increasing RH compared with the second peak values, as shown in Figure 9a. The small current at the first peak implies that insufficient protons are supplied into the membrane. This might be due to the lack of water on the membrane, which in turn is because of relatively strong hydrophobicity caused by the relatively small ionic channel density. Also, the development of the ionic channel network might be insufficient due to small water on the membrane. Thus, the first peak value denotes relatively low ionic clusters and ionic channel network density. This assumption explains the small current variation of the first peak with increasing RH. 

By a similar assumption, the second peak may be attributed to high ionic cluster and ionic channel network density. The increasing current value of the second peak might be connected with the enhancement of the ionic channel network. The peak current at 75% RH is 10 nA and is five times higher value than the peak current at 45% RH. This result implies that the developed ionic channel network allows more than five times higher the number of protons to move in the high-current region. The ratio of the first and second peak is similar at all RH conditions. This might be connected with the membrane health information. The small ratio means a relatively poor developed ionic channel network density and an otherwise relatively well-developed ionic channel network. At all RH values, the ratio is approximately 20. It is very difficult to conclude that the membrane is healthy; however, it can be used as an indicator for the comparison of the relative membrane health. 

Figure 9b shows the FWHM variation under a high current and depicts the uniformity of the ionic channel network in the membrane. The proton movement into the membrane depends on the uniformity of the ionic channel network distribution. When the network is uniformly developed, proton movement into the membrane might be even and the current shows an equal distribution. Thus, narrow and wide FWHM represent uniform and non-uniform ionic channel network distribution on the membrane, respectively. The FWHM of the histograms enlarges with increasing RH, as shown in Figure 9b, which indicates an increase in the uniformity of the ionic channel network. Through hydration, the ionic channel network develops non-uniformly in the membrane. This development depends on the density of ionic channels. When water content on the membrane is increased, the development of the ionic channel network is proportional to the ionic channel density in the membrane. Thus, the uniformity of the ionic channel network increases through hydration. 

For a more detailed understanding, the peak and mean current values under different RH conditions have been numerically analyzed. For this, several things have been assumed. First, the protons move into the membrane similarly to how electrons move into the external load. Second, the protons, while moving into the ionic channel, maintain a steady state while the tip is scanning. From basic principles of electromagnetism, the current in the conductor is defined as the total charge value during the measurement time, as shown in Equation (2).

(2) $$I=\frac{dQ}{dt}$$

where dQ and dt are charge and time, respectively. The total charge Q is defined as

(3) Q=∫Idt

Additionally, Q is given by the multiplication of the number of protons moving into the ionic channel network (NPMI) and the basic charge value, which are denoted by N and e, respectively. Thus, Equation (3) becomes

(4) Ne=∫Idt

From Equation (3), the number of charge carriers is derived and used in Equation (5).

(5) $$N=\frac{\int Idt}{e}$$

When the current (I) is assumed in a steady state, it is independent of time and the integration with respect to time is the same as the total time. Then, Equation (5) becomes

(6) $$N=\frac{I\Delta t}{e}$$

Total time Δt is related to the scan rate. From the basic principles of AFM, the scan rate is reciprocal with the scanning time for a series of pixels, which is expressed as one over the scan rate. Δt is the total time for which the tip stops at a pixel. Thus, total time Δt is given by

(7) $$\Delta t=\frac{\frac{1}{v}}{p}=\frac{1}{pv}$$

where v and p are scan rate and number of pixels, respectively. Therefore, the number of protons moving through the ionic channel network can be expressed as:

(8) $$N=\frac{I}{pve}$$

NPMI is directly connected to the ionic channel network density. When the ionic channel network density is high, various proton movement pathways exist in the membrane. Thus, the number of protons obtained from Equation (8) might be proportional to the ionic channel density. It is not easy to determine the morphology of the ionic channel network in the membrane, but Equation (8) provides indirect numerical information about the ionic channel density. If the NPMI differs by a factor of two in randomly selected positions on the membrane, the ionic channel network densities beneath these positions are also presumed to differ by the factor of two. Therefore, Equation (8) can be used for the quantitative analysis of the ionic channel network. 

**Figure 10:**
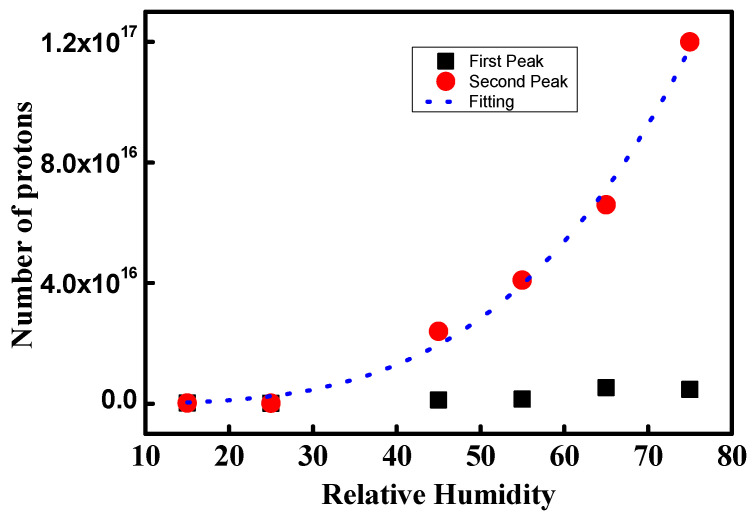
NPMI for the low and high ionic channel density regions.

Figure 10 shows NPMI in the low and high ionic channel density regions. In dry conditions, NPMI is 2.0 × 10^14^ and 2.9 × 10^14^ in the two regions. Under hydration, the NPMI in the low-current region is 10 times higher than that in dry conditions. The NPMI at 45% and 55% RH has similar values of 1.2 × 10^15^ and 1.5 × 10^15^, respectively. At 65% and 75%, the number of protons increases as 5.3 × 10^15^ and 4.7 × 10^15^, respectively. Through hydration from 55% to 65% RH, the NPMI increases drastically. However, between 65% and 75% RH, no increase in the number of protons can be observed. This implies that the development of the ionic channel network is limited due to low ionic channel density.

NPMI calculated from the second peaks shows a different trend compared to that from the first peaks. NPMI increases continuously as 2.4 × 10^16^, 4.1 × 10^16^, 6.6 × 10^16^, and 1.2 × 10^17^ at 45%, 55%, 65%, and 75% RH, respectively. Also, the increase in NPMI becomes swifter with increasing hydration. At 75% RH, the NPMI is 50 times larger compared with that at the dry condition. This indicates that the proton pathway increases by approximately 50 times. Thus, a 50-times-larger ionic channel network density is developed compared with the dry condition for the second peak. The dotted blue line in Figure 10 is the fitted line of the second peak and gives numerical information about the increasing rate of the ionic channel network density. It follows a power series, such as:

y = 3x^3.5^ (9)

where x and y are RH and ionic channel density, respectively. 

**Figure 11:**
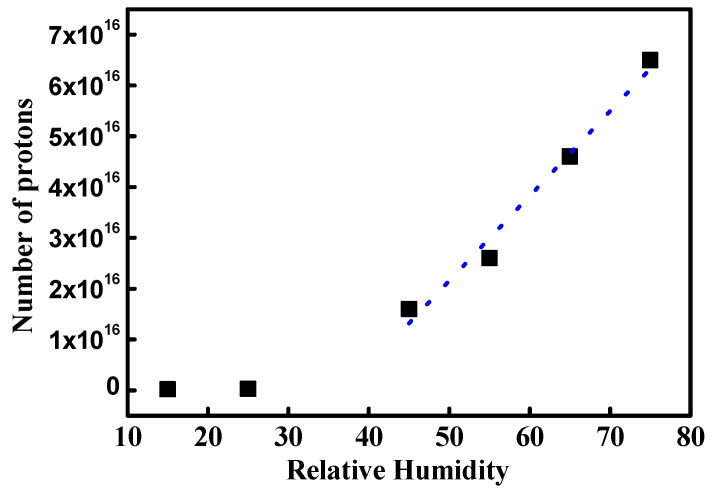
Mean NPMI under different RH conditions. Blue dot is fitted line of high RH condition.

Figure 11 shows the mean NPMI under different RH conditions and depicts the average ionic channel density. NPMI at low and high RH shows discontinuous variation. At 45% RH, NPMI is 1.6 × 10^16^, which is 55 times larger than that in dry conditions. This result implies that proton movement increases 55 times due to drastic ionic channel network development. Thus, it can be concluded that the ionic channel network might have increased by 55 times. At increasing RH values, NPMIs are 2.6 × 10^16^, 4.6 × 10^16^, and 6.5 × 10^16^ at 55%, 65%, and 75%, respectively. The dotted blue line in Figure 10 is the fitted line of mean NPMI. It follows a linear curve with a slope of 1.67 × 10^15^. This indicates the increasing rate of ionic channel network density under hydration. In other words, the linearly increasing NPMI might be due to the linearly developing ionic channel network under hydration. It is difficult to directly verify the increase in ionic channel network under hydration. One way to roughly verify the same is by using increments in proton conductivity at different RH conditions. Table 3 shows the linearly increasing proton conductivity of Nafion with increasing RH, as reported by other research groups. The data are in good agreement with our results. The differences between the mean and second peak NPMI are 30%, 32%, 43%, and 46% at 45%, 55%, 65%, and 75% RH, respectively. This difference is expressed as the uniformity of ionic channel density. The rising difference with increasing RH indicates a reduced uniformity of the ionic channel network. This result implies that the ionic channel network development by hydration is drastically increased; however, the local rate of increase is different at different positions on the membrane.

## 5. Conclusions

In this study, the morphological change in the ionic channel network in Nafion under different RH conditions is analyzed through a developed quantitative approximation model based on CSAFM images. The quantitative approximation model is derived from calculating charge carriers into a sample. CSAFM measures a weak current between a conductive tip and the sample surface. Based on electrodynamics, the number of charge carriers can be calculated from the electrical current. Based on this principle, the simple model for calculating the number of protons moving into the ionic channel network can be derived in this study. The number of protons moving into the ionic channel network is proportional to the ionic channel network density. Thus, the model might be used for analyzing the ionic channel network variation in the PEM.

For a quantitative understanding of ionic channel density variation under different RH, NPMIs have been calculated from the model. Mean NPMI, which gives the overall ionic channel density on the membrane, increased drastically on changing the condition from dry to hydration. On increasing RH, the NPMI increased linearly with a rate that might be identical to the development of the ionic channel network under hydration. The difference between the second peak of the histogram, which is extracted from the CSAFM image, and mean NPMI denotes the uniformity of the ionic channel network density. The difference between the second peak and NPMI enlarged after increasing hydration. Thus, it can be concluded that the uniformity of the ionic channel network decreased with hydration.

## Figures and Tables

**Table 1:** RMS roughness of Nafion 115 at each RH.

RH	17	27	45	55	65	75
RMS roughness	18.3 (7.0)	8.82	19.5	21.9	20.1	21.4

**Table 2:** Peak value and FWHM of each current map histogram.

RH	Average	1st Peak		2nd Peak	
		Peak (nA)	FWHM (nA)	Peak (nA)	FWHM (nA)
15%	0.016	0.016	0.0012	None	None
25%	0.024	0.012	0.0035	None	None
45%	1.28	0.1	0.04	2	0.56
55%	2.11	0.12	0.048	3.36	1.96
65%	3.77	0.44	0.34	5.4	5.6
75%	5.35	0.39	1.38	10	None

**Table 3:** Proton conductivity at different RH conditions reported in the literature.

RH (%)		30	40	50	60	70	80	90	References
**Proton Conductivity (Scm^−1^)**	Nafion	0.06	0.07	0.08	0.094	0.10	0.12		[28]
	Recast Nafion		0.002	0.005	0.008		0.015	0.022	[29]
	Nafion 117	0.075		0.1		0.12	0.14		[30]
	Nafion 117	0.05		0.07		0.1	0.12		[31]

## Data Availability

Data are contained within the article.

## References

[1] Sharma S., Bruno G. (2012). Pollet Support Materials for PEMFC and DMFC Electrocatalysts-A Review. J. Power Source.

[2] Ge S.-H., Yi B.-L. (2003). A Mathematical Model for PEMFC in Different Flow Modes. J. Power Sources.

[3] Ouyang M., Xu L., Li J., Lu L., Gao D., Xie Q. (2006). Performance Comparison of Two Fuel Cell Hybrid Buses with Different Powertrain and Energy Management Strategies. J. Power Source.

[4] Wilhelm A.N., Surgenor B.W., Pharoah J.G. (2006). Design and Evaluation of a Micro-Fuel-Cell-Based Power System for a Mobile Robot. IEEE ASME Trans. Mechatron..

[5] Kamarudin S.K., Achmad F., Daud W.R.W. (2009). Overview on the Application of Direct Methanol Fuel Cell (DMFC) for Portable Electronic Devices. Int. J. Hydrogen Energy.

[6] Mauritz K.A., Moore R.B. (2004). State of Understanding of Nafion. Chem. Rev..

[7] Hsu W.Y., Gierke T.D. (1983). Ion Transport and Clustering in Nafion Perfluorinated Membranes. J. Membr. Sci..

[8] Fujimura M., Hashimoto T., Kawai H. (1981). Small-Angle X-ray Scattering Study of Perfluorinated Ionomer Membranes. 1. Origin of Two Scattering Maxima. Micromolecules.

[9] Rollet A.-L., Diat O., Gebel G. (2002). A New Insight into Nafion Structure. J. Phys. Chem. B.

[10] Schmidt-Rohr K., Chen Q. (2008). Parallel Cylindrical Water Nanochannels in Nafion Fuel-Cell Membranes. Nat. Mater..

[11] Duvigneau J., Schonherr H., Vancso G.J. (2010). Nanoscale Thermal AFM of Polymers: Transient Heat Flow Effects. ACS Nano.

[12] Sen S., Subramanian S., Discher D.E. (2005). Indentation and Adhesive Probing of a Cell Membrane with AFM: Theoretical Model and Experiments. Biophys. J..

[13] Xie X., Kwon O., Zhu D.M., Van Nguyen T.V., Lin G.Y. (2007). Local Probe and Conduction Distribution of Proton Exchange Membranes. J. Phys. Chem. B.

[14] Melin T., Diesinger H., Deresmes D., Stievenard D. (2004). Electric Force Microscopy of Individually Charged Nanoparticles on Conductors: An Analytical Model for Quantitative Charge Imaging. Phys. Rev. B.

[15] Han B., Chang J.X., Song W., Sun Z., Yin C.Q., Lv P.H., Wang X. (2019). Study on Micro Interfacial Charge Motion of Polyethylene Nanocomposite Based on Electrostatic Force Microscope. Polymers.

[16] Deschler J., Seiler J., Kindersberger J. (2017). Detection of Charges at the Interphase of Polymeric Nanocomposites. IEEE Trans. Dielect. Electr. Insul..

[17] Shen Y., Wang Y., Zhou Y., Hai C.X., Hu J., Zhang Y. (2018). Electrostatic Force Spectroscopy Revealing the Degree of Reduction of Individual Graphene Oxide Sheets. Beilstein J. Nanotechnol..

[18] Barnes A.M., Buratto S.K. (2018). Imaging Channel Connectivity in Nafion Using Electrostatic Force Microscopy. J. Phys. Chem. B.

[19] Yi S., Zhang F., Li W., Huang C., Zhang H., Pan M. (2011). Anhydrous Elevated-Temperature Polymer Electrolyte Membranes Based on Ionic Liquids. J. Membr. Sci..

[20] Barnes A.M., Du Y., Liu B., Zhang W., Seifert S., Coughlin E.B., Buratto S.K. (2019). Effect of Surface Alignment on Connectivity in Phosphonium-Containing Diblock Copolymer Anion-Exchange Membranes. J. Phys. Chem. C.

[21] Wang X., Habte B.T., Zhang S., Yang H., Zhao J., Jiang F., He Q. (2019). Localized Electrochemical Impedance Measurements on Nafion Membranes: Observation and Analysis of Spatially Diverse Proton Transport Using Atomic Force Microscopy. Anal. Chem..

[22] He Q., Ren X. (2012). Scanning Probe Imaging of Surface Ion Conductance in an Anion Exchange Membrane. J. Power Source.

[23] Kwon O., Kang Y., Wu S., Zhu D.M. (2010). Characteristics of Microscopic Proton Current Flow Distributions in Fresh and Aged Nafion Membranes. J. Phys. Chem. B.

[24] Kang Y., Kwon O., Xie X., Zhu D.-M. (2009). Conductance Mapping of Proton Exchange Membranes by Current Sensing Atomic Force Microscopy. J. Phys. Chem. B.

[25] Hiesgen R., Helmly S., Galm I., Morawietz T., Handl M., Friedrich K.A. (2012). Microscopic Analysis of Current and Mechanical Properties of Nafion^®^ Studied by Atomic Force Microscopy. Membranes.

[26] Hara M., Daiki H., Inukai J., Hara M., Miyatake K., Watanabe M. (2014). Reversible/Irreversible Increase in Proton-Conductive Areas on Proton-Exchange-Membrane Surface by Applying Voltage using Current-Sensing Atomic Force Microscope. J. Electroanal. Chem..

[27] Son B., Park J., Kwon O. (2021). Analysis of Ionic Domains on a Proton Exchange Membrane Using a Numerical Approximation Model Based on Electrostatic Force Microscopy. Polymers.

[28] Sharma P.P., Kim D. (2022). A Facile and Sustainable Enhancement of Anti-Oxidation Stability of Nafion Membrane. Membranes.

[29] Chien H.-C., Tsai L.-D., Huang C.-P., Kang C.-Y., Lin J.-N., Chang F.-C. (2013). Sulfonated Graphene Oxide/Nafion Composite Membranes for High-Performance Direct Methanol Fuel Cells. Int. J. Hydrogen Energy.

[30] Wang C., Li N., Shin D.W., Lee S.Y., Kang N.R., Lee Y.M., Guiver M.D. (2011). Fluorene-Based Poly(arylene ether sulfone)s Containing Clustered Flexible Pendant Sulfonic Acids as Proton Exchange Membranes. Macromolecules.

[31] Huang Y.C., Tai R.H., Lee H.F., Wang R.H., Gopal R., Lee C.C., Chang M.Y., Huang W.Y. (2016). Synthesis of Highly Sulfonated Poly(arylene ether) Containing Multiphenyl for Proton Exchange Membrane Materials. Int. J. Polym. Sci..

